# Simultaneous Production of a Virus-Like Particle Linked to dsRNA to Enhance dsRNA Delivery for Yellow Head Virus Inhibition

**DOI:** 10.3390/v14122594

**Published:** 2022-11-22

**Authors:** Jaruwan Worawittayatada, Kitipong Angsujinda, Rapee Sinnuengnong, Pongsopee Attasart, Duncan R. Smith, Wanchai Assavalapsakul

**Affiliations:** 1 Department of Microbiology, Faculty of Science, Chulalongkorn University, Bangkok 10330, Thailand; 2 Aquatic Resources Research Institute, Chulalongkorn University, Bangkok 10330, Thailand; 3 Department of Research and Development, Queen Saovabha Memorial Institute, The Thai Red Cross Society, Bangkok 10330, Thailand; 4 Center of Applied Shrimp Research and Innovation, Institute of Molecular Biosciences, Mahidol University, Nakorn Pathom 73170, Thailand; 5 Institute of Molecular Biosciences, Mahidol University, Nakorn Pathom 73170, Thailand

**Keywords:** yellow head virus, virus-like particle, ds*pro*, *Penaeus stylirostris* densovirus, inhibition, shrimp

## Abstract

A co-expressed *Penaeus stylirostris* densovirus (*Pst*DNV) capsid and dsRNA specific to the yellow head virus (YHV) *protease* (CoEx cp*Pst*DNV/ds*pro*) has been shown to suppress YHV replication in the Pacific white-legged shrimp (*Litopenaeus vannamei*). However, maintaining two plasmids in a single bacterial cell is not desirable; therefore, a single plasmid harboring both the *Pst*DNV capsid and the dsRNA-YHV-*pro* gene was constructed under the regulation of a single T7 promoter, designated pET28a-Linked cp*Pst*DNV-ds*pro*. Following induction, this novel construct expressed an approximately 37-kDa recombinant protein associated with a roughly 400-bp dsRNA (Linked cp*Pst*DNV-ds*pro*). Under a transmission electron microscope, the virus-like particles (VLP; Linked *Pst*DNV VLPs-ds*pro*) obtained were seen to be monodispersed, similar to the native *Pst*DNV virion. A nuclease digestion assay indicated dsRNA molecules were both encapsulated and present outside the Linked *Pst*DNV VLPs-ds*pro*. In addition, the amount of dsRNA produced from this strategy was higher than that obtained with a co-expression strategy. In a YHV infection challenge, the Linked *Pst*DNV VLPs-ds*pro* was more effective in delaying and reducing mortality than other constructs tested. Lastly, the linked construct provides protection for the dsRNA cargo from nucleolytic enzymes present in the shrimp hemolymph. This is the first report of a VLP carrying virus-inhibiting dsRNA that could be produced without disassembly and reassembly to control virus infection in shrimp.

## 1. Introduction

According to the Global Aquaculture Alliance (GAA), shrimp aquaculture is one of the fastest-growing food production sectors [1]. From the early 1990s to the present, Thailand has been one of the largest shrimp exporters amongst Asian countries [2,3]. However, pervasive disease outbreaks have become a significant problem in Pacific white-legged shrimp (*Litopenaeus vannamei*) farming, as witnessed by substantial production declines and fluctuation of yearly income [4]. Yellow head virus (YHV) is the causative agent of yellow head disease (YHD) which affects a number of shrimp species [5]. YHV infection of shrimp can result in up to 100% mortality within 3 to 10 days from the start of an outbreak [5,6]. YHV is classified in the genus *Okavirus*, Family *Roniviridae* in the Order *Nidovirales*. The YHV virion has an enveloped bacilliform shape with dimensions of 40–60 × 150–200 nm, which contains a positive-sense single-stranded RNA (+ssRNA) genome [7,8,9].

RNA interference (RNAi) is an innate immune mechanism which can be applied to reduce specific viral protein synthesis via mRNA–dsRNA interactions [10,11]. Briefly, long or short dsRNA in the cell will be cleaved by the RNase III Dicer into small interfering RNAs (siRNAs) of approximately 22 nucleotides. The siRNA becomes part of the multicomponent RNA-induced silencing complex (RISC) and is subsequently unwound to single-stranded RNA. The anti-sense RNA is retained in the RISC and used as a guide to target the viral mRNA. Finally, the mRNA target is degraded, leading to viral suppression and a decrease in shrimp mortality. Therefore, RNAi is a potentially powerful therapeutic strategy to suppress the replication of target DNA and RNA viruses in shrimp [12,13]. Previous studies have shown that dsRNA of the YHV *protease* (*pro*) gene could be a potential target for reducing YHV replication in shrimp [14,15,16,17].

A variety of strategies have been used to carry dsRNA into shrimp. Synthetic chitosan and liposomes have been shown to be potential candidates for dsRNA delivery. The positive charged polymers, namely cationic polysaccharide chitosan, and the cholesterol-based cationic liposome gave a promising protective efficacy toward virus infection [18,19]. Furthermore, oral administration of food with engineered unicellular organisms expressing dsRNA such as *E. coli* [20], probiotics (*Lactobacillus plantarum*) [21], and microalgae [22,23] have all been investigated, and all could reduce shrimp mortality upon virus challenge. However, the applications of genetically modified organisms in shrimp species that are food species are concerning owing to the risk of environmental gene transfer. However, to prevent the enzymatic degradation and promote the uptake of protective dsRNA, improvements to the specificity and efficacy of the current delivery methods are necessary.

Virus-like particles (VLPs) have been developed as a biological nanocarrier system as they are structurally identical to the native virion and specific to the host species but are non-infectious. VLPs can self-assemble as a consequence of the expression of the capsid protein of naked viruses, and these VLPs can protect nucleic acids from host enzymatic digestion [24,25]. For shrimp viruses, it has been previously shown that expression of the *Penaeus stylirostris* densovirus (*Pst*DNV) capsid gene in a bacterial system resulted in a self-assembled VLP of approximately 37 kDa [26,27]. Similarly, *Macrobrachium rosenbergii* nodavirus (*Mr*NV) capsid proteins can form a VLP that could be disassembled and reassembled in a calcium-dependent manner allowing for the encapsulation of both DNA and dsRNA [28,29,30]. While different bacterial hosts are normally used for the expression of the dsRNA and the VLP, Wuthisathid and colleagues recently developed a novel *E. coli* host optimized to be able to express both molecules [31]. Interestingly, Sinnuengnong et al. [32] reported that the co-expression of the *Pst*DNV capsid protein (cp*Pst*DNV) and dsRNA-YHV-*pro* in the same *E. coli* cell generated VLP that reduced YHV replication and shrimp mortality at a higher level than just administration of the dsRNA. However, two plasmids, encoding the viral capsid and dsRNA, are difficult to be continuously maintained in the same bacteria cell, as one of the two plasmids can be subsequently lost due to incompatibility.

The purpose of this research was to develop a plasmid with the *Pst*DNV capsid gene linked to the dsRNA-YHV-*pro* (ds*pro*) construct under the control of a single T7 RNA polymerase promoter to improve the preventive efficacy against YHV infection by regulating the expression level of the two molecules (*Pst*DNV capsid and ds*pro*). The expression, purification, and characterization of the two molecules was undertaken and the efficacy of the recombinant linked cp*Pst*DNV-ds*pro* in suppressing YHV was tested in Pacific white-legged shrimp.

## 2. Materials and Methods

### 2.1. Recombinant Bacteria and Plasmid Extraction

*Escherichia coli* strain DH5α containing either a pET-17b plasmid containing two double-stranded RNAs which encode the ribonucleotide reductase small subunit (*rr2*) gene of white spot syndrome virus and the *protease* (*pro*) gene of yellow head virus (pET17b-ds*rr2*-ds*pro*_one stem) [33], or a pET-28a(+) plasmid encoding the *Pst*DNV capsid (pET28a-cp*Pst*DNV) [32] was inoculated into Luria-Bertani (LB) broth supplemented with 100 µg/mL ampicillin or 50 µg/mL kanamycin, respectively. The bacteria were incubated at 37 °C overnight with vigorous shaking.

To isolate the plasmids, the bacterial cells were harvested by centrifugation at 8500× *g* for 2 min at ambient temperature and resuspended in STET buffer [8% (*w*/*v*) sucrose, 0.1% (*v*/*v*) Triton X-100, 50 mM EDTA and 50 mM Tris, pH 8.0]. Lysozyme was added to the solution, which was then incubated at 37 °C for 10 min, and boiled at 100 °C for 45 sec. The supernatant was then collected following centrifugation at 18,890× *g* for 15 min at 4 °C. Five percent (*w*/*v*) Cetyltrimethylammonium bromide (CTAB) was added after removing the cell debris and the solution was centrifuged at 18,890× *g* for 20 min at 4 °C and the supernatant was discarded. The plasmid DNA pellet was dissolved in 1.2 M NaCl containing 10 µg/mL RNase A (New England Biolabs, Ipswich, MA, USA) followed by incubation at 37 °C for 30 min. An equal volume of chloroform was added and mixed and the solution was centrifuged at 18,890× *g* for 15 min at 4 °C. The aqueous phase was transferred into a new microcentrifuge tube and precipitated with an equal volume of isopropanol at −20 °C for 1 h. Then, the solution was centrifuged at 18,890× *g* for 15 min at 4 °C. The pellet was then washed with 75% (*v*/*v*) ethanol and centrifuged at 18,890× *g* for 10 min at 4 °C. Finally, the pellet was dried, dissolved in distilled sterile DEPC-treated water, and analyzed by agarose gel electrophoresis.

### 2.2. Construction and Expression of a Recombinant Linked cpPstDNV-Dspro Plasmid

Following the digestion of pET17b-ds*rr2*-ds*pro*_one stem by *Xho*I (Thermo Fisher Scientific, Waltham, MA, USA), the short stem dsRNA of the *protease* (*pro*) gene was cloned into pre-digested pET28a-cp*Pst*DNV expression plasmid at the same restriction site. The recombinant plasmid (pET28a-Linked cp*Pst*DNV-ds*pro*) was transformed into *E. coli* strain DH5α. The recombinant clones were verified by selective medium (LB agar containing 50 µg/mL kanamycin), rapid size screening, restriction enzyme digestion, and DNA sequencing (Bioneer, Daejeon, South Korea). The primers used for pET28a-Linked cp*Pst*DNV-ds*pro* sequencing are listed in Table 1.

pET28a-Linked cp*Pst*DNV-ds*pro* with the correct nucleotide sequence was transformed into *E. coli* strain Rosetta-gami 2(DE3) pLysS for expression. A single colony of the recombinant bacteria was cultured at 30 °C in LB medium containing 34 and 50 µg/mL of chloramphenicol and kanamycin, respectively. When the culture reached 0.4 OD_600 nm_, isopropyl-β-D-thiogalactopyranoside (IPTG) was added to the culture at 0.4 mM final concentration. After incubation at 30 °C for 3 h, the bacterial cells were harvested by centrifugation at 18,890× *g* for 10 min at 4 °C. Proteins of a portion of the induced cells were analyzed by SDS– polyacrylamide gel electrophoresis (PAGE) and western blotting with a His Tag Antibody (R&D System Inc., Minneapolis, MN, USA). In parallel, the expression of dsRNA was confirmed by PAGE following total RNA isolation using TRIzol reagent (Thermo Fisher Scientific, Waltham, MA, USA) and nuclease digestion assay.

### 2.3. Purification of Recombinant Linked cpPstDNV-Dspro by Affinity Chromatography

Induced bacteria (150 OD_600 nm_) containing pET28a-Linked cp*Pst*DNV-ds*pro* were resuspended in ENZhance Lysis Buffer (NSTDA, Pathum Thani, Thailand). The supernatant was then collected, and the volume increased to 5 mL with binding buffer [0.5 M NaCl and 20 mM Tris-HCl, pH 8.0], and purified as described by Sinnuengnong and colleagues with minor modification [32]. In brief, the solution was loaded onto a Zn^2+^ saturated HisTrap HP column (Cytiva, Marlborough, MA, USA). The column was washed with a washing buffer (binding buffer containing 40 mM imidazole) and sample was eluted with an elution buffer (binding buffer containing 300 mM imidazole). The remaining protein was removed from the column with a stripping buffer (binding buffer containing 50 mM EDTA). Fractions were analyzed by SDS-PAGE and western blotting. The elution buffer was later changed to a binding buffer using an Amicon^®^ Ultra-4 Centrifugal Filter Unit (Merck Millipore, Darmstadt, Germany) or selected fractions. Protein concentrations were determined using a Bio-Rad protein assay kit (BIO-RAD Laboratories, Hercules, CA, USA) using bovine serum albumin (BSA) as a standard. Fractions containing purified protein were pooled and stored at 4 °C. 

### 2.4. Characterization of Recombinant Linked cpPstDNV-Dspro

To determine whether the recombinant Linked cp*Pst*DNV-ds*pro* could form virus-like particles, the concentrated protein was stained with 1% (*v*/*v*) phosphotungstic acid and visualized under a transmission electron microscope (TEM) (JEOL JEM-1400, Tokyo, Japan) at the Scientific and Technological Research Equipment Centre (STREC), Chulalongkorn University, Thailand. 

After virus-like particle formation (Linked *Pst*DNV VLPs-ds*pro*) was confirmed by TEM, to verify whether ds*pro* was present on and/or in the Linked *Pst*DNV VLPs-ds*pro*, total RNA isolation was direct conducted from a concentrated protein, then treated with RNase A, RNase III or DNase I for 30 min at 37 °C. In addition, the external dsRNA was removed from concentrated protein by incubation with RNase III (New England Biolabs, Ipswich, MA, USA) at 37 °C for 30 min prior to total RNA isolation by TRIzol reagent and subjected to a nuclease digestion assay. All RNA samples were later analyzed by PAGE.

To examine the stability of recombinant Linked *Pst*DNV VLPs-ds*pro*, the concentrated protein was kept at 4 °C for 6 months. At the indicated time point, Linked *Pst*DNV VLPs-ds*pro* was analyzed by TEM as described above.

### 2.5. Effect of Recombinant Linked PstDNV VLPs-Dspro in YHV Infected L. vannamei

*L. vannamei* (approximate size of 300–400 mg) were obtained from a local farm in Nakorn Pathom province, Thailand. *L. vannamei* were raised at 28 °C in plastic aquaria containing 10 ppt saline water. During a 7-day acclimation, shrimp were randomly sampled to determine any infection with virus, bacteria, or ectoparasites according to lab routine procedure. 

*L. vannamei* were divided into five experimental groups (10–13 shrimps/group) for injection with (1) 150 mM NaCl, (2) Naked ds*pro* (30 ng dsRNA), (3) *Pst*DNV VLPs (22 μg protein), (4) co-expressed *Pst*DNV VLPs/ds*pro* (CoEx *Pst*DNV VLPs/ds*pro*; 30 ng dsRNA and 22 μg protein) [32], and (5) Linked *Pst*DNV VLPs-ds*pro* (30 ng dsRNA and 13 μg protein) [Note: the purified either *Pst*DNV VLPs, CoEx *Pst*DNV VLPs/ds*pro* or Linked *Pst*DNV VLPs-ds*pro* was diluted with 150 mM NaCl, while naked ds*pro* was extracted from *E. coli* HT115 and directly resuspended in 150 mM NaCl. All of them were then prepared to the required concentration with 150 mM NaCl prior to injection into shrimp]. At the appropriate time point, all groups were challenged with YHV at dilution that gives a 100% shrimp death on day 3 post-infection by hemocoel injection. Shrimp mortality was counted daily for 7 days. The cumulative mortality was compared by Newman-Keuls multiple comparison tests. A *p*-value < 0.05 was considered as statistically significant.

At the end of the experiment, all shrimps were euthanized by chilling on ice after which the gill tissue of every individual was collected and total RNAs were isolated and subjected to RT-PCR to confirm the shrimp were infected with YHV. YHV infection was confirmed by semi-quantitative RT-PCR with a set of *helicase-*specific primers (Table 1). Briefly, cDNA was synthesized from 2 μg of total RNA with random hexamers and RevertAid reverse transcriptase (Thermo Fisher Scientific, Waltham, MA, USA) following the manufacturer’s instructions. Multiplex PCR amplification was undertaken in a reaction containing 1 μL of cDNA, 1 × PCR buffer, 2 mM MgCl_2_, 0.2 mM dNTP mix, 0.2 μM YHV-Helicase-F primer, 0.2 μM YHV-Helicase-R primer, 0.2 μM of Actin-F primer, 0.2 μM Actin-R primer, and 1 unit of *Taq* DNA polymerase (biotechrabbit, Berlin, Germany). Thermal cycling was performed as follows: 94 °C for 2 min, followed by 25 cycles of 94 °C for 10 sec, 55 °C for 30 sec, 72 °C for 60 sec. The PCR products were analyzed by electrophoresis through 2.0% TAE-buffered agarose gel. 

### 2.6. Hemolymph Enzymatic Digestion

To determine whether the Linked *Pst*DNV VLPs-ds*pro* complex could protect dsRNA from shrimp enzymes, an enzyme digestion assay was performed. Hemolymph was collected from 10 shrimp (approximately 1–2 g weight) and gently mixed on ice with binding buffer [0.5 M NaCl and 20 mM Tris-HCl, pH 8.0] at a 2:1 ratio. Then, the complete homogenate was centrifuged at 8500× *g* for 10 min at 4 °C to remove any left-over shrimp tissue. The supernatant was incubated with recombinant Linked *Pst*DNV VLPs-ds*pro* at a ratio of 1:1 (45 ng of total RNA/reaction) at 28 °C for 3 and 6 h. At the indicated time points, total RNA was isolated by TRIzol reagent and analyzed by PAGE.

## 3. Results

### 3.1. Construction and Expression of Recombinant pET28a-Linked cpPstDNV-Dspro

**Figure 1:**
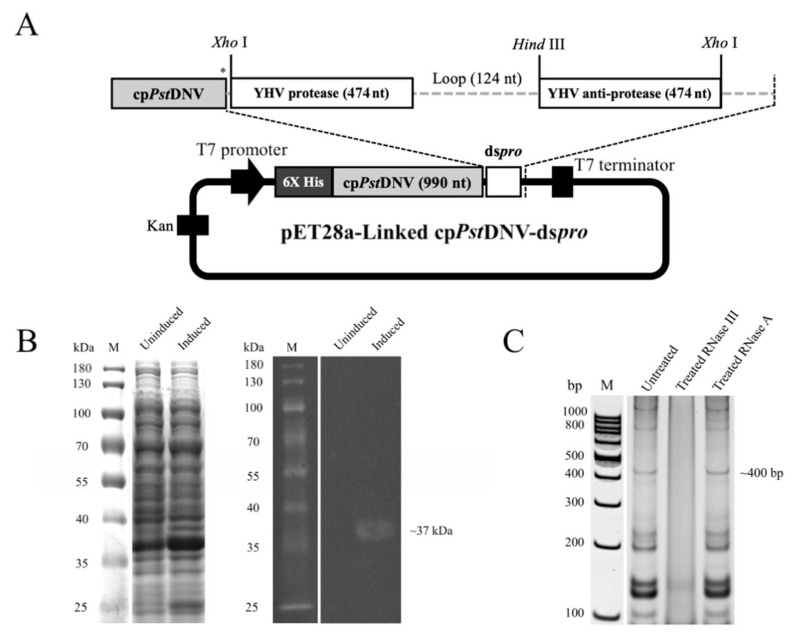
Construction of recombinant pET28a-Linked cp*Pst*DNV-ds*pro* vector and expression of its counterpart. (**A**) Schematic illustration for recombinant pET28a-Linked cp*Pst*DNV-ds*pro*. Asterisk indicates a stop codon. (**B**) SDS-PAGE and western blot analysis of total cell lysate from induced recombinant *E. coli* strain Rosetta-gami 2(DE3) pLysS. Lane M, PageRuler™ Prestained Protein Ladder (Thermo Fisher Scientific, Waltham, MA, USA). (**C**) The dsRNA-YHV *protease* gene expression and its properties. Total RNA isolated from induced bacterial cells was treated with RNase III or RNase A and analyzed on 10% TAE buffered-polyacrylamide gel. An arrow indicates ds*pro*. Lane M, GeneRuler 100 bp DNA Ladder (Thermo Fisher Scientific, Waltham, MA, USA).

In this study, the short stem dsRNA of the *protease* (*pro*) gene was successfully cloned into the pET28a-cp*Pst*DNV expression vector at the *Xho*I site (Figure 1A). DNA sequencing verified the recombinant Linked cp*Pst*DNV-ds*pro* plasmid, confirming the presence of the *Pst*DNV capsid gene [32] and the dsRNA specific to *pro* gene of the YHV genome (GenBank accession no. FJ848675.1; located at 8710–9183 nt), respectively. The nucleotide sequence of Linked cp*Pst*DNV-ds*pro* plasmid was shown to have 100% homo-logy with the two reference sequences (Supplementary Figure S1).

The recombinant protein was successfully expressed in *E. coli* strain Rosetta-gami 2(DE3) pLysS after induction with a final concentration of 0.4 mM IPTG at 30 °C for 3 h. The total cell lysates were analyzed by SDS-PAGE and western blotting. The results showed the expected recombinant protein band of approximately 37 kDa (Figure 1B), which was the same expected size as seen in induced cell lysates of cp*Pst*DNV alone and co-expressed cp*Pst*DNV/ds*pro* (CoEx cp*Pst*DNV/ds*pro*) (Supplementary Figure S2A). To determine the expression of the dsRNA of the *pro* gene, total RNA was extracted from the induced cells prior to incubation with RNase III or RNase A at 37 °C for 30 min. The analysis on an acrylamide gel showed an RNA band at approximately 400 bp (Figure 1C), corresponding to the ds*pro* complementarily binding of the sense and anti-sense strands of the YHV *protease* gene (~470 nucleotides each). The band was completely digested by the dsRNA-specific RNase III, but not by the single-stranded RNA specific RNase A. The identical RNA patterns were also observed in CoEx cp*Pst*DNV/ds*pro* and induced pET17b-ds*pro* in the *E. coli* HT115 strain, which lacks RNase III activity (Supplementary Figure S2B).

### 3.2. Purification of Recombinant pET28a-Linked cpPstDNV-Dspro

**Figure 2:**
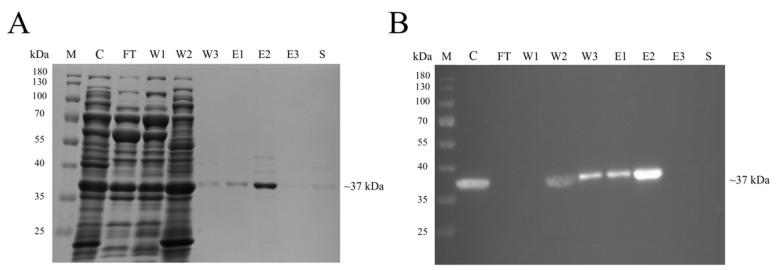
Purification of the recombinant Linked cp*Pst*DNV-ds*pro* by affinity chromatography. (**A**) SDS-PAGE and (**B**) western blot analysis of fractions collected from a Zn^2+^ saturated HisTrap HP column (Cytiva, Marlborough, MA, USA). The expected band size of Linked cp*Pst*DNV-ds*pro* was approximately at 37 kDa. Lane M, PageRuler™ Prestained Protein Ladder (Thermo Fisher Scientific, Waltham, MA, USA); Lane C, crude extract solution; Lane FT, flow thought solution; Lanes W1–W3, proteins eluted by washing buffer; Lanes E1–E3, proteins eluted by elution buffer; Lane S, proteins eluted by stripping buffer.

One hundred and fifty OD_600 nm_ of induced recombinant cells was lysed in ENZhance Lysis Buffer. The supernatant was filtered using a 0.45-µm membrane and subsequently loaded onto a Zn^2+^-saturated HisTrap HP column. The eluted protein fraction clearly showed the presence of the 37-kDa protein band in SDS-PAGE, and the band was also detected in a western blot using an anti-His Tag antibody (Figure 2).

### 3.3. Characterization of Recombinant Linked cpPstDNV-Dspro

**Figure 3:**
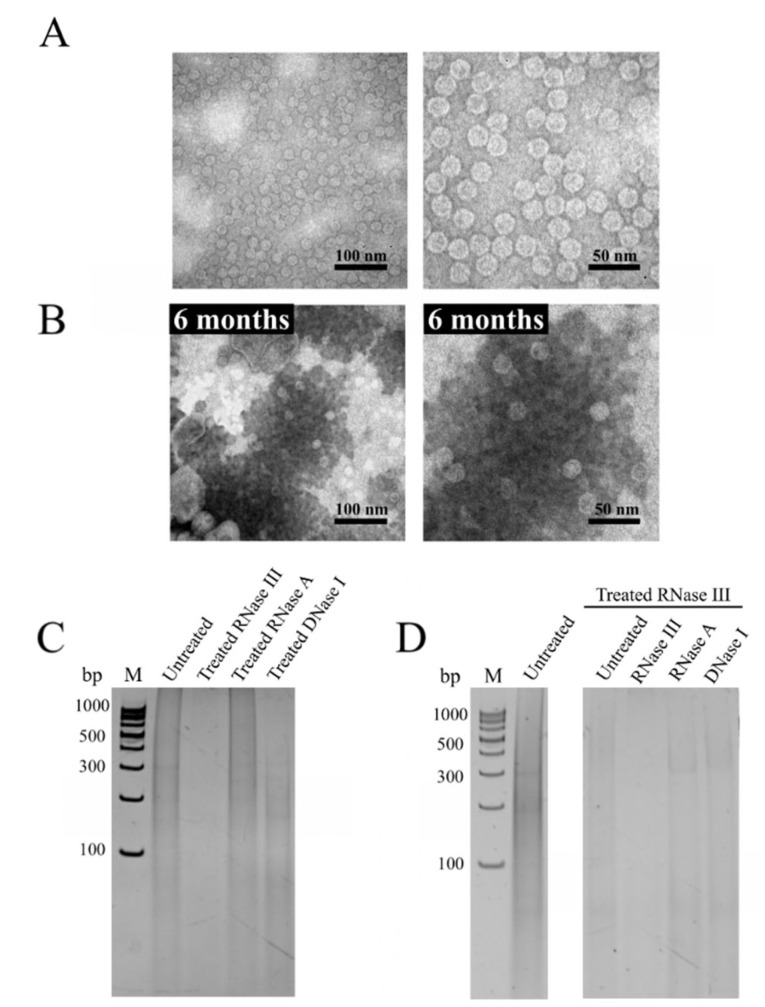
Characterization of recombinant Linked cp*Pst*DNV-ds*pro* counterpart. (**A**) Transmission electron micrograph of Linked cp*Pst*DNV-ds*pro* (Linked *Pst*DNV VLPs-ds*pro*). (**B**) Linked *Pst*DNV VLPs-ds*pro* after 6-month storage at 4 °C. (**C**) The presence of dsRNA on the outer surface of Linked *Pst*DNV VLPs-ds*pro*. Total RNA isolated from purified recombinant VLP was directly treated with either RNase III, RNase A or DNase I. (**D**) The encapsulated dsRNA in Linked *Pst*DNV VLPs-ds*pro*. Intrinsic dsRNA was confirmed by RNA isolation of RNase III-treated Linked *Pst*DNV VLPs-ds*pro*, followed by incubation with either RNase III, RNase A, or DNase I. All RNA products were analyzed on a 12% TAE buffered-polyacrylamide gel. Lane M, GeneRuler 100 bp DNA Ladder (Thermo Fisher Scientific, Waltham, MA, USA).

To obtain detailed visual information on VLP formation, the purified recombinant Linked cp*Pst*DNV-ds*pro* was analyzed by TEM. The electron microscopy showed monodispersed icosahedral *Pst*DNV-like particles (Linked *Pst*DNV VLPs-ds*pro*) with an approximate diameter of 20–30 nm (Figure 3A). To investigate the presence of dsRNA (ds*pro*) on or in the Linked *Pst*DNV VLPs-ds*pro*, total RNA was extracted from the purified VLPs which was then incubated with RNase III, RNase A, or DNase I. Following PAGE, the results showed that extracted RNA (~20 to 800 bp in size) was completely digested by RNase III, but not by RNase A or DNase I (Figure 3C). A similar RNA pattern was observed when Linked *Pst*DNV VLPs-ds*pro* was treated with RNase III before total RNA extraction and nuclease digestion (Figure 3D). This finding demonstrates that ds*pro* is displayed on the surface and encapsulated in the Linked *Pst*DNV VLPs-ds*pro.* TEM analysis showed the packaging of ds*pro* did not affect the assembly of the *Pst*DNV capsid, which maintained the correct structural form. The amount of extracted RNAs was approximately 57.7 ng/µg of purified Linked *Pst*DNV VLPs-ds*pro,* showing 10-fold more dsRNA entrapped within the nanoparticles. In addition, Linked *Pst*DNV VLPs-ds*pro* remained intact after 6 months of storage at 4 °C (Figure 3B); both *Pst*DNV VLPs and CoEx cp*Pst*DNV VLPs/ds*pro* also showed long term stability of the particles (Supplementary Figure S3).

### 3.4. Inhibition of YHV Infection of L. vannamei by Linked PstDNV VLPs-Dspro

**Figure 4:**
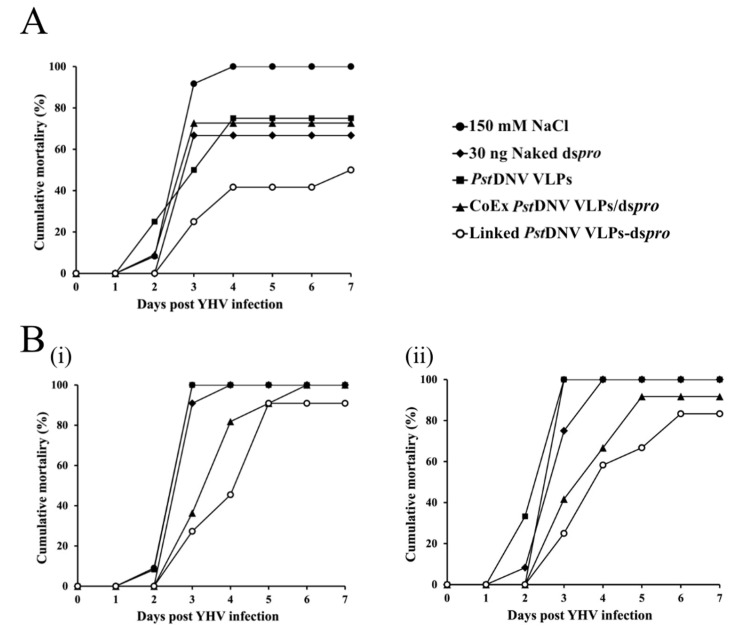
Inhibitory effect of Linked *Pst*DNV VLPs-ds*pro* against YHV infection in *L. vannamei*. Cumulative mortality of shrimp (n = 10−13) treated with 150 mM NaCl, naked ds*pro*, *Pst*DNV VLPs, CoEx *Pst*DNV VLPs/ds*pro* or Linked *Pst*DNV VLPs-ds*pro* for 5 (**A**) or 7 (**B**) days prior to challenge with YHV at dilution that gives a 100% shrimp death on day 3 post infection by hemocoel injection. [Note: i and ii are two independent experiments].

To determine whether Linked *Pst*DNV VLPs-ds*pro* could inhibit YHV infection *L. vannamei*, shrimp were injected into the hemolymph with either naked ds*pro*, *Pst*DNV VLPs, CoEx *Pst*DNV VLPs/ds*pro,* Linked *Pst*DNV VLPs-ds*pro*, or 150 mM NaCl. On day 5 post-injection, shrimp were challenged with YHV, and mortality was monitored daily for 7 days. The cumulative mortality of shrimp at day 7 post-challenge following treatment with different constructs is shown in Figure 4A. A 100% cumulative shrimp mortality was seen in the negative control group (150 mM NaCl) at 4 days post challenge, whereas those groups that received either naked ds*pro*, *Pst*DNV VLPs, or CoEx *Pst*DNV VLPs/ds*pro* presented approximately 70% cumulative mortality after YHV infection. Interestingly, shrimp treated with Linked *Pst*DNV VLPs-ds*pro* showed a reduction of cumulative mortality to 50%, indicating that the Linked *Pst*DNV VLPs-ds*pro* could protect the therapeutic ds*pro*. The RT-PCR products on agarose gels confirmed that shrimp in all groups were infected with YHV (Supplementary Figure S4). In additional experiments, the pre-viral challenge period was increased to 7 days, and results showed that Linked *Pst*DNV VLPs-ds*pro*-injected shrimp had a cumulative mortality of 80–90%, which was lower than the 100% mortality seen in all other groups (Figure 4B). This indicated that the ds*pro* entrapped in the *Pst*DNV VLPs was more effective than all other treatments in viral suppression and in reducing the mortality of shrimp.

### 3.5. Hemolymph Enzymatic Digestion Assay

**Figure 5:**
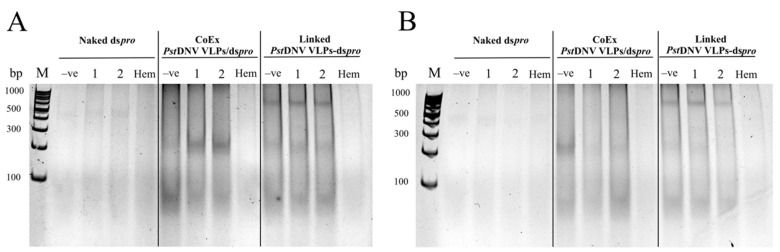
dsRNA protective efficiency of Linked *Pst*DNV VLPs-ds*pro* from hematopoietic enzyme degradation in shrimp. Total RNA isolated from Linked *Pst*DNV VLPs-ds*pro* was analyzed on a 12% TAE buffered-polyacrylamide gel following an incubation with hemolymph at 28 °C for (**A**) 3 and (**B**) 6 h. Lane M, GeneRuler 100 bp DNA Ladder (Thermo Fisher Scientific, Waltham, MA, USA); Lane −ve, unincubated with hemolymph; Lane 1–2, incubated with hemolymph (duplicate experiments); Lane Hem, total RNA extracted from shrimp hemolymph.

To verify the protective functionality of Linked *Pst*DNV VLPs-ds*pro*, the RNA of the purified Linked *Pst*DNV VLPs-ds*pro* was further investigated after incubation with hemolymph, the shrimp circulatory fluid that has nucleolytic digestion enzymes. At 6 h post incubation, total RNA was extracted from CoEx *Pst*DNV VLPs/ds*pro* was partially enzymatically degraded more than that from Linked *Pst*DNV VLPs-ds*pro*, as a fainter RNA band was observed. In addition, naked ds*pro* extracted from *E. coli* strain HT115 was detected after mixing with hemolymph at 3 h, with a marked decrease in total RNA after 6 h of incubation (Figure 5). Hence, this experiment demonstrated that both groups (Linked *Pst*DNV VLPs-ds*pro* and CoEx *Pst*DNV VLPs/ds*pro*) showed efficacy in protecting dsRNA from enzymatic digestion.

## 4. Discussion

While many viruses can cause disease in farmed shrimp [34], two shrimp viruses, namely white spot syndrome virus and yellow head virus, are the major causes of economic loss to the shrimp aquaculture industry in many Asian countries, including Thailand [34,35]. Previous research has reported that a range of molecules including natural compounds, recombinant proteins, virus-like particles (VLPs), DNA vaccines, and specific double-stranded RNAs could all suppress viral replication or prevent viral infection in shrimp [13,36]. Amongst these, the application of specific double-stranded RNAs is a powerful strategy to suppress endogenous genes [10,37,38] or viral gene expression [14,15,33,39] to significantly inhibit viral replication in vitro and in vivo in shrimp. Recently, the co-expression of *Pst*DNV capsid and dsRNA-YHV-*pro* was applied to protect shrimp from YHV infection [32]. However, the transcription from two plasmids carrying different genes could not be equally regulated, and there can be difficulties in maintaining two plasmids in the same cell over the long term [40]. 

This study reports the production of a recombinant *Pst*DNV VLP coupled with a short stem-loop dsRNA of the YHV *protease* gene under the same promoter in a prokaryotic expression system. We aimed to remove the need to freshly prepare double transformants, as a single plasmid can be easily maintained in a bacterial cell. In this study, the YHV *protease* gene was inserted into a pET-28a(+) plasmid harboring the *Pst*DNV capsid gene at its 3′ end. Following induction with IPTG, the recombinant Linked cp*Pst*DNV-ds*pro* of roughly 37 kDa could be expressed in *E. coli* strain Rosetta-gami 2(DE3) pLysS and assembled to form VLPs in a solubilizing agent. The icosahedral particles obtained are similar to native virions [41] and recombinant *Pst*DNV VLPs produced in *E. coli* [26,27,32] and insect cell [42]. Surprisingly, Linked *Pst*DNV VLPs-ds*pro* was dispersed as individually free particles. This indicated that the ds*pro* and hexa-histidine tag did not interfere with VLP formation, and this strategy could avoid the conjugation of VLPs, which affects dsRNA delivery [32]. TEM analysis further showed Linked *Pst*DNV VLPs-ds*pro* remained stable after being kept at 4 °C for 6 months. This result is possibly due to a ‘jelly roll’ fold, an element of the *Pst*DNV capsid that makes this viral structure stable at a wide range of pH, as well as possessing resistance to hydrolysis, and long-term stability [27,43]. 

According to the nuclease digestion assay, ds*pro* was found both on the outside and encapsulated in the Linked *Pst*DNV VLPs-ds*pro*. Based on the ratio of ds*pro* to cp*Pst*DNV, the amount of ds*pro* obtained from our new strategy was higher than that from a co-expression strategy. This confirmed an increased yield of dsRNA and could reduce the production cost of the recombinant molecules when transcription is regulated by only one promoter. Similar to the co-expression strategy, ds*pro* of Linked *Pst*DNV VLPs-ds*pro* was protected from enzymatic digestion by the shrimp hemolymph. However, when shrimp were treated with Linked *Pst*DNV VLPs-ds*pro* and other constructs followed by YHV challenge, shrimp receiving Linked *Pst*DNV VLPs-ds*pro* showed the lowest cumulative mortality amongst those treated with CoEx *Pst*DNV VLPs/ds*pro*, *Pst*DNV alone, ds*pro* alone, or NaCl, suggesting that the Linked *Pst*DNV VLPs-ds*pro* could effectively shield dsRNA from host nuclease and immune response, thus assisting in endocytosis and successful delivery. The protection toward therapeutic nucleotides of *Pst*DNV VLP and *Mr*NV VLP have been already demonstrated both in vitro and in vivo in shrimp [27,28,29,30,32,44,45,46]. Ds*pro* alone could reduce shrimp’s mortality from YHV infection, but at a comparatively lower rate. Yodmuang and colleagues [15] showed the inhibitory effect of ds*pro* toward YHV could last for at least 5 days post injection in *Penaeus monodon*. Similarly, *Pst*DNV VLPs alone could reduce shrimp mortality, presumably due to a triggering of the shrimp innate immunity via binding of *Pst*DNV which is a pathogen-associated molecular pattern to the host Toll-like receptor [47,48]. An oral administration of these treatments should be investigated in a further study to assess a feasible use in the shrimp farming industry.

Linked cp*Pst*DNV-ds*pro* produced in this study was partially solubilized and most recombinant molecules accumulate in a host cell as inclusion bodies (Supplementary Figure S5) as a consequence of lack of host cell post-translational modifications [49,50], and a similar observation was found for CoEx cp*Pst*DNV/ds*pro* and cp*Pst*DNV alone [32]. Lower induction temperature and longer incubation time may reduce the problem [51], the plasmid might be co-expressed with molecular chaperones, or a fusion protein or a signal peptide could be added [50,52,53,54] to enhance the solubility of the Linked cp*Pst*DNV-ds*pro* in the future. 

## 5. Conclusions

We demonstrated the development of *Pst*DNV VLPs transporting dsRNA-*pro* produced from the recombinant *Pst*DNV capsid gene linked to the dsYHV-*protease* gene could inhibit yellow head virus infection of *L. vannamei*. Given the higher RNA concentration of the dsRNA as compared to the co-expressed product after the same procedure, our complex product would potentially be suitable for mass production. 

## Figures and Tables

**Table 1:** Oligonucleotides used this study.

**Primers**	**Sequences (5′–3′)**	**Purposes**
T7	TAATACGACTCACTATAGGG	pET28a-Linked cp*Pst*DNV-ds*pro* sequencing
T7 terminator	GCTAGTTATTGCTCAGCGG	
Internal cp*Pst*DNV F1	GATGTGTCGCAAGTTTGGTG	
Internal cp*Pst*DNV F2	CAACTAAGGAAGCCGACGTAACATTGG	
Internal cp*Pst*DNV R	CATCCCCAAACTTGCGACACATC	
YHV-Helicase-F	CAAGGACCACCTGGTACCGGTAAGAC	semi-quantitative RT-PCR for YHV-Helicase
YHV-Helicase-R	GCGGAAACGACTGACGGCTACATTCAC	
Actin-F	ATGGCATCTCGCAAGAAGATT	Internal control for semi-quantitative RT-PCR
Actin-R	TTAGCAAGAGCATGCATCCTG	

## Data Availability

The data used to support the findings of this study are included within the article.

## Supplementary Materials

The following supporting information can be downloaded at: https://www.mdpi.com/article/10.3390/v14122594/s1, Figure S1: Nucleotide sequence alignment of pET28a-Linked cp*Pst*DNV-ds*pro* with other relevant plasmid and gene; Figure S2: Expression of recombinant Linked cp*Pst*DNV-ds*pro*, cp*Pst*DNV and CoEx cp*Pst*DNV/ds*pro* in *E. coli* strain Rosetta-gami 2(DE3) pLysS; Figure S3: Transmission electron micrograph of *Pst*DNV VLPs, CoEx *Pst*DNV VLPs/ds*pro* and Linked *Pst*DNV VLPs-ds*pro* and their stability after being kept at 4 °C for 6 months; Figure S4: Confirmation of shrimp in all treatments infected with YHV; Figure S5: The solubility of recombinant Linked cp*Pst*DNV-ds*pro* in ENZhance lysis buffer.
