# Supplementary material for: Simultaneous Production of a Virus-Like Particle Linked to dsRNA to Enhance dsRNA Delivery for Yellow Head Virus Inhibition
Source: Viruses. 2022 Nov 22;14(12):2594. doi: 10.3390/v14122594 (PMC9785521; doi:10.3390/v14122594)
Supplement: Supplementary file 1 [file viruses-14-02594-s001.zip › viruses-2028738-supplementary.pdf]

viruses

MDPI

Supplementary Materials

```
                                 1                                                                              80
cpPstDNV Sinnuengnong  (1)       ATGGGCAGCAGCCATCATCATCATCATCACAGCAGCGGCCTGGTGCCGCGCGGCAGCCATATGTGCGCCGATTCAACAAG
cpPstDNV-dspro         (1)       ATGGGCAGCAGCCATCATCATCATCATCACAGCAGCGGCCTGGTGCCGCGCGGCAGCCATATGTGCGCCGATTCAACAAG
FJ848675 YHV protease  (1)       --------------------------------------------------------------------------------
YHV anti protease      (1)       --------------------------------------------------------------------------------
                                 81                                                                            160
cpPstDNV Sinnuengnong  (81)      AGCAAGCCCAAGGAAAAGATCCAGGAGGGATGCACATAATGAAGACGAAGAACACGCCGAAGGATCAAGTGGACCAGACC
cpPstDNV-dspro         (81)      AGCAAGCCCAAGGAAAAGATCCAGGAGGGATGCACATAATGAAGACGAAGAACACGCCGAAGGATCAAGTGGACCAGACC
FJ848675 YHV protease  (1)       --------------------------------------------------------------------------------
YHV anti protease      (1)       --------------------------------------------------------------------------------
                                 161                                                                           240
cpPstDNV Sinnuengnong  (161)     CACACAGATGTCTACAATTCAATACTGGAGACTCAATATATATTACTTTCCAAACAAGAAGATACTTCGAATTCGACGCT
cpPstDNV-dspro         (161)     CACACAGATGTCTACAATTCAATACTGGAGACTCAATATATATTACTTTCCAAACAAGAAGATACTTCGAATTCGACGCT
FJ848675 YHV protease  (1)       --------------------------------------------------------------------------------
YHV anti protease      (1)       --------------------------------------------------------------------------------
                                 241                                                                           320
cpPstDNV Sinnuengnong  (241)     GCCAATGATGGAAACTTCGACGGAAAAAATTTATACTGCCTCCCACTACATTGGATGAACTTATATCTCTATGGTCTAAA
cpPstDNV-dspro         (241)     GCCAATGATGGAAACTTCGACGGAAAAAATTTATACTGCCTCCCACTACATTGGATGAACTTATATCTCTATGGTCTAAA
FJ848675 YHV protease  (1)       --------------------------------------------------------------------------------
YHV anti protease      (1)       --------------------------------------------------------------------------------
                                 321                                                                           400
cpPstDNV Sinnuengnong  (321)     GAGCAGCGACAGTTCAGCAACAGAAACACAACGATATAAGATGGTAAAATCAATGATGAAGACCTACGGATGGAAAGTAC
cpPstDNV-dspro         (321)     GAGCAGCGACAGTTCAGCAACAGAAACACAACGATATAAGATGGTAAAATCAATGATGAAGACCTACGGATGGAAAGTAC
FJ848675 YHV protease  (1)       --------------------------------------------------------------------------------
YHV anti protease      (1)       --------------------------------------------------------------------------------
                                 401                                                                           480
cpPstDNV Sinnuengnong  (401)     ATAAAGCAGGCGTAGTGATGCACTCGATGGTACCCCTTATGAAAGACTTAAAAGTATCAGGAGGCACATCATTTGAGACT
cpPstDNV-dspro         (401)     ATAAAGCAGGCGTAGTGATGCACTCGATGGTACCCCTTATGAAAGACTTAAAAGTATCAGGAGGCACATCATTTGAGACT
FJ848675 YHV protease  (1)       --------------------------------------------------------------------------------
YHV anti protease      (1)       --------------------------------------------------------------------------------
                                 481                                                                           560
cpPstDNV Sinnuengnong  (481)     CTCACATTTACAGACACCCCATATTTAGAAATATTTAAGGATACTACTGGACTACATAATCAACTATCAACTAAGGAAGC
cpPstDNV-dspro         (481)     CTCACATTTACAGACACCCCATATTTAGAAATATTTAAGGATACTACTGGACTACATAATCAACTATCAACTAAGGAAGC
FJ848675 YHV protease  (1)       --------------------------------------------------------------------------------
YHV anti protease      (1)       --------------------------------------------------------------------------------
                                 561                                                                           640
cpPstDNV Sinnuengnong  (561)     CGACGTAACATTGGCAAAATGGATACAAAATCCCCAACTTGTGACCGTACAATCAACAGCAGCAAACTATGAAGACCCAA
cpPstDNV-dspro         (561)     CGACGTAACATTGGCAAAATGGATACAAAATCCCCAACTTGTGACCGTACAATCAACAGCAGCAAACTATGAAGACCCAA
FJ848675 YHV protease  (1)       --------------------------------------------------------------------------------
YHV anti protease      (1)       --------------------------------------------------------------------------------
                                 641                                                                           720
cpPstDNV Sinnuengnong  (641)     TCCAACAATTTGGATTCATGGAACAAATGCGAACCGGTGACAGAAAAGCCTATACAATCCATGGTGACACTAGAAATTGG
cppstDNV_dspro01       (641)     TCCAACAATTTGGATTCATGGAACAAATGCGAACCGGTGACAGAAAAGCCTATACAATCCATGGTGACACTAGAAATTGG
FJ848675 YHV protease  (1)       --------------------------------------------------------------------------------
YHV anti protease      (1)       --------------------------------------------------------------------------------
                                 721                                                                           800
cpPstDNV Sinnuengnong  (721)     TATGGCGGAGAAATACCAACAACCGGACCCACCTTCATCCCAAAATGGGGTGGTCAATTAAAATGGGACAAACCATCCCT
cpPstDNV-dspro         (721)     TATGGCGGAGAAATACCAACAACCGGACCCACCTTCATCCCAAAATGGGGTGGTCAATTAAAATGGGACAAACCATCCCT
FJ848675 YHV protease  (1)       --------------------------------------------------------------------------------
YHV anti protease      (1)       --------------------------------------------------------------------------------
                                 801                                                                           880
cpPstDNV Sinnuengnong  (801)     TGGAAACCTAGTCTACCCAGCAGACCACCATACAAACGACTGGCAACAGATCTTCATGAGAATGTCACCAATCAAAGGAC
cpPstDNV-dspro         (801)     TGGAAACCTAGTCTACCCAGCAGACCACCATACAAACGACTGGCAACAGATCTTCATGAGAATGTCACCAATCAAAGGAC
FJ848675 YHV protease  (1)       --------------------------------------------------------------------------------
YHV anti protease      (1)       --------------------------------------------------------------------------------
                                 881                                                                           960
cpPstDNV Sinnuengnong  (881)     CAAATGGAGACGAACTTAAACTTGGCTGCAGAGTACAAGCCGACTTCTTCCTACATCTAGAAGTACGACTCCCACCACAA
cpPstDNV-dspro         (881)     CAAATGGAGACGAACTTAAACTTGGCTGCAGAGTACAAGCCGACTTCTTCCTACATCTAGAAGTACGACTCCCACCACAA
FJ848675 YHV protease  (1)       --------------------------------------------------------------------------------
YHV anti protease      (1)       --------------------------------------------------------------------------------
                                 961                                                                          1040
cpPstDNV Sinnuengnong  (961)     GGATGTGTCGCAAGTTTGGGGATGTTACAATATCTTCACGCACCATGTACTGGACAACTTAACAAATGTTATATTATGCA
cpPstDNV-dspro         (961)     GGATGTGTCGCAAGTTTGGGGATGTTACAATATCTTCACGCACCATGTACTGGACAACTTAACAAATGTTATATTATGCA
FJ848675 YHV protease  (1)       --------------------------------------------------------------------------------
YHV anti protease      (1)       --------------------------------------------------------------------------------
                                 1041           XhoI                                                          1120
cpPstDNV Sinnuengnong  (1041)    TACTAACTAA----------------------------------------------------------------------
cpPstDNV-dspro         (1041)    TACTAACTAATAACTCGAGTCAGCGGCAAATTCCTCTACTTTCCTCGTCACATCTTCGACTCCTGTACTGACAACACTCT
FJ848675 YHV protease  (1)       ------------CTCGAGTCAGCGGCAAATTCCTCTACTTTCCTCGTCACATCTTCGACTCCTGTACTGACAACACTCT
YHV anti protease      (1)       --------------------------------------------------------------------------------
                                 1121                                                                         1200
cpPstDNV Sinnuengnong  (990)     --------------------------------------------------------------------------------
cpPstDNV-dspro         (1121)    CACACGACACATCCGCGTCACAAAAGGTGAAGAAACTCATGACATCGAATTGTTGAGCGAAGAATATGACGCCACTCCTT
FJ848675 YHV protease  (68)      CACACGACACATCCGCGTCACAAAAGGTGAAGAAACTCATGACATCGAATTGTTGAGCGAAGAATATGACGCCACTCCTT
YHV anti protease      (1)       --------------------------------------------------------------------------------
```

```
                                   1201                                                                          1280
cpPstDNV Sinnuengnong  (990)    --------------------------------------------------------------------------------
cpPstDNV-dspro         (1201)   TCATCAAAACCGACAGTCCATTCGCAGAAGCAACTGTACTCAAATTCGGTAAACTCCAACGCACTCAGTACGCATACTTC
FJ848675 YHV protease  (148)    TCATCAAAACCGACAGTCCATTCGCAGAAGCAACTGTACTCAAATTCGGTAAACTCCAACGCACTCAGTACGCATACTTC
YHV anti protease      (1)      --------------------------------------------------------------------------------
                                   1281                                                                          1360
cpPstDNV Sinnuengnong  (990)    --------------------------------------------------------------------------------
cpPstDNV-dspro         (1281)   GTCACTGCTGATGACATCAGGGTTGGTTCAATGTCCGCCGACGGCTACCACAACATTTCTACCAAGGATGGTGACTGCGG
FJ848675 YHV protease  (228)    GTCACTGCTGATGACATCAGGGTTGGTTCAATGTCCGCCGACGGCTACCACAACATTTCTACCAAGGATGGTGACTGCGG
YHV anti protease      (1)      --------------------------------------------------------------------------------
                                   1361                                                                          1440
cpPstDNV Sinnuengnong  (990)    --------------------------------------------------------------------------------
cpPstDNV-dspro         (1361)   TTCACTCCTCTTTGACCACCTTCACAATGTTGTTGGAGCTCACATCGTCGGCATTGCTAGCATCCCTCCTGTTAACGGTG
FJ848675 YHV protease  (308)    TTCACTCCTCTTTGACCACCTTCACAATGTTGTTGGAGCTCACATCGTCGGCATTGCTAGCATCCCTCCTGTTAACGGTG
YHV anti protease      (1)      --------------------------------------------------------------------------------
                                   1441                                                                          1520
cpPstDNV Sinnuengnong  (990)    --------------------------------------------------------------------------------
cpPstDNV-dspro         (1441)   CCCTGACCTGGAATGCAGAAAAGGAAATGCTCTGCGGACCAAATGATGACTACGATTACGATCCAGAAAAAGTCGGTCCA
FJ848675 YHV protease  (388)    CCCTGACCTGGAATGCAGAAAAGGAAATGCTCTGCGGACCAAATGATGACTACGATTACGATCCAGAAAAAGTCGGTCCA
YHV anti protease      (1)      --------------------------------------------------------------------------------
                                   1521                              loop                                        1600
cpPstDNV Sinnuengnong  (990)    --------------------------------------------------------------------------------
cpPstDNV-dspro         (1521)   CCCAAGGTATGGCCTGTAGAATCAATCACTGCTCTCAGCACGATCCTCAATCAGCTCAAGTATGTCACCGGAGATGCCTT
FJ848675 YHV protease  (468)    CCCAAGGTATGGC-------------------------------------------------------------------
YHV anti protease      (1)      --------------------------------------------------------------------------------
                                   1601                   loop                                                   1680
cpPstDNV Sinnuengnong  (990)    --------------------------------------------------------------------------------
cpPstDNV-dspro         (1601)   CACTACACCCAAACTCCTTACAAACTATCAGCTAATTGGTTGCGAGACACTGGACCAAGCTTGCCATACCTTGGGTGGAC
FJ848675 YHV protease  (480)    --------------------------------------------------------------------------------
YHV anti protease      (1)      --------------------------------------------------------------GCCATACCTTGGGTGGAC
                                   1681                                                                          1760
cpPstDNV Sinnuengnong  (990)    --------------------------------------------------------------------------------
cpPstDNV-dspro         (1681)   CGACTTTTCTGGATCGTAATCGTAGTCATCATTTGGTCCGCAGAGCATTTCCTTTTCTGCATTCCAGGTCAGGGCACCGT
FJ848675 YHV protease  (480)    --------------------------------------------------------------------------------
YHV anti protease      (18)     CGACTTTTCTGGATCGTAATCGTAGTCATCATTTGGTCCGCAGAGCATTTCCTTTTCTGCATTCCAGGTCAGGGCACCGT
                                   1761                                                                          1840
cpPstDNV Sinnuengnong  (990)    --------------------------------------------------------------------------------
cpPstDNV-dspro         (1761)   TAACAGGAGGGATGCTAGCAATGCCGACGATGTGAGCTCCAACAACATTGTGAAGGTGGTCAAAGAGGAGTGAACCGCAG
FJ848675 YHV protease  (480)    --------------------------------------------------------------------------------
YHV anti protease      (98)     TAACAGGAGGGATGCTAGCAATGCCGACGATGTGAGCTCCAACAACATTGTGAAGGTGGTCAAAGAGGAGTGAACCGCAG
                                   1841                                                                          1920
cpPstDNV Sinnuengnong  (990)    --------------------------------------------------------------------------------
cpPstDNV-dspro         (1841)   TCACCATCCTTGGTAGAAATGTTGTGGTAGCCGTCGGCGGACATTGAACCAACCCTGATGTCATCAGCAGTGACGAAGTA
FJ848675 YHV protease  (480)    --------------------------------------------------------------------------------
YHV anti protease      (178)    TCACCATCCTTGGTAGAAATGTTGTGGTAGCCGTCGGCGGACATTGAACCAACCCTGATGTCATCAGCAGTGACGAAGTA
                                   1921                                                                          2000
cpPstDNV Sinnuengnong  (990)    --------------------------------------------------------------------------------
cpPstDNV-dspro         (1921)   TGCGTACTGAGTGCGTTGGAGTTTACCGAATTTGAGTACAGTTGCTTCTGCGAATGGACTGTCGGTTTTGATGAAAGGAG
FJ848675 YHV protease  (480)    --------------------------------------------------------------------------------
YHV anti protease      (258)    TGCGTACTGAGTGCGTTGGAGTTTACCGAATTTGAGTACAGTTGCTTCTGCGAATGGACTGTCGGTTTTGATGAAAGGAG
                                   2001                                                                          2080
cpPstDNV Sinnuengnong  (990)    --------------------------------------------------------------------------------
cpPstDNV-dspro         (2001)   TGGCGTCATATTCTTCGCTCAACAATTCGATGTCATGAGTTTCTTCACCTTTTGTGACGCGGATGTGTCGTGTGAGAGTG
FJ848675 YHV protease  (480)    --------------------------------------------------------------------------------
YHV anti protease      (338)    TGGCGTCATATTCTTCGCTCAACAATTCGATGTCATGAGTTTCTTCACCTTTTGTGACGCGGATGTGTCGTGTGAGAGTG
                                   2081                                                      XhoI
cpPstDNV Sinnuengnong  (990)    ------------------------------------------------------------   2140
cpPstDNV-dspro         (2081)   TTGTCAGTACAGGAGTCGAAGATGTGACGAGGAAAGTAGAGGAATTTGCCGCTGATCGAG   2140
FJ848675 YHV protease  (480)    ------------------------------------------------------------   2140
YHV anti protease      (418)    TTGTCAGTACAGGAGTCGAAGATGTGACGAGGAAAGTAGAGGAATTTGCCGCTGATCGAG   2140
```

Figure S1. Nucleotide sequence alignment of pET28a-Linked cp*Pst*DNV-ds*pro* with other relevant plasmid and gene.

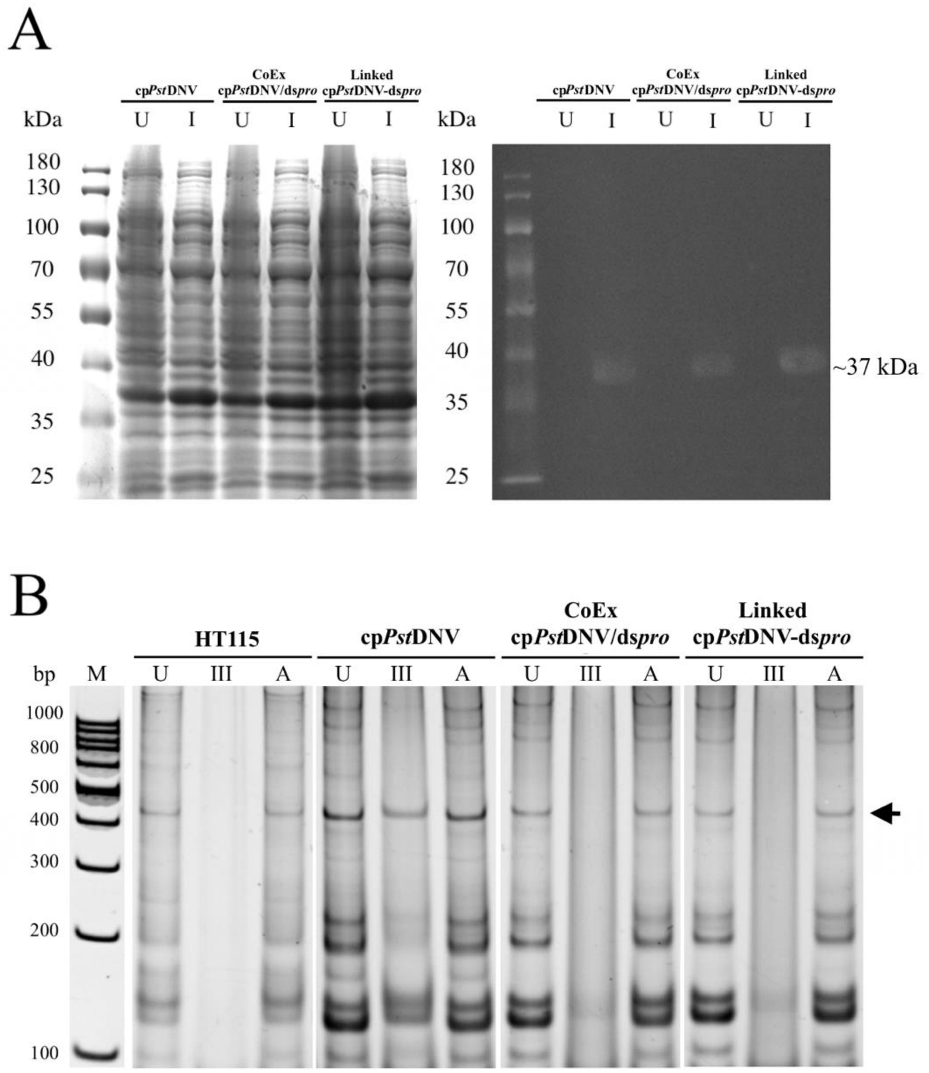

Figure S2. Expression of recombinant Linked cp*Pst*DNV-ds*pro*, cp*Pst*DNV and CoEx cp*Pst*DNV/ds*pro* in *E. coli* strain Rosetta-gami 2(DE3) pLysS. (A) SDS-PAGE and western blot analysis from the total cell lysate of induced (lane I) and uninduced (U) recombinant bacteria. Lane M, PageRuler™ Prestained Protein Ladder (Thermo Fisher Scientific, Waltham, MA, USA) (B) The dsRNA-YHV *protease* gene expression and its properties were analyzed on 12% TAE buffered-polyacrylamide gel after total RNAs isolated from induced recombinant *E. coli* strain Rosetta-gami 2(DE3) pLysS and HT115 (lane U) were treated with RNase III (lane III) or RNase A (lane A). Lane M, GeneRuler 100 bp DNA Ladder (Thermo Fisher Scientific, Waltham, MA, USA). Arrow indicates ds*pro*.

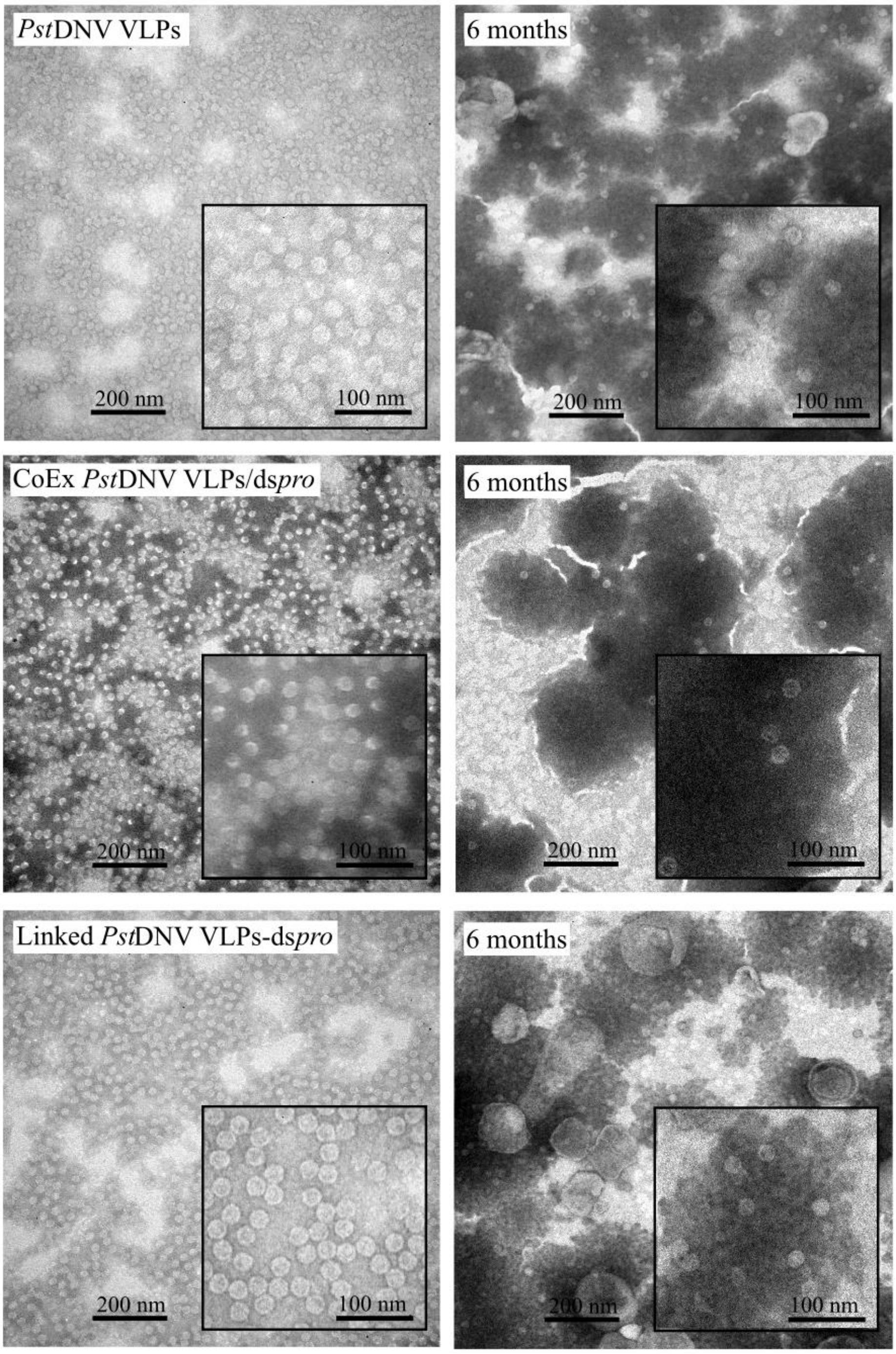

Figure S3. Transmission electron micrograph of *Pst*DNV VLPs, CoEx *Pst*DNV VLPs/ds*pro* and Linked *Pst*DNV VLPs-ds*pro* and their stability after being kept at 4 °C for 6 months. Bars = 200 and 100 nm.

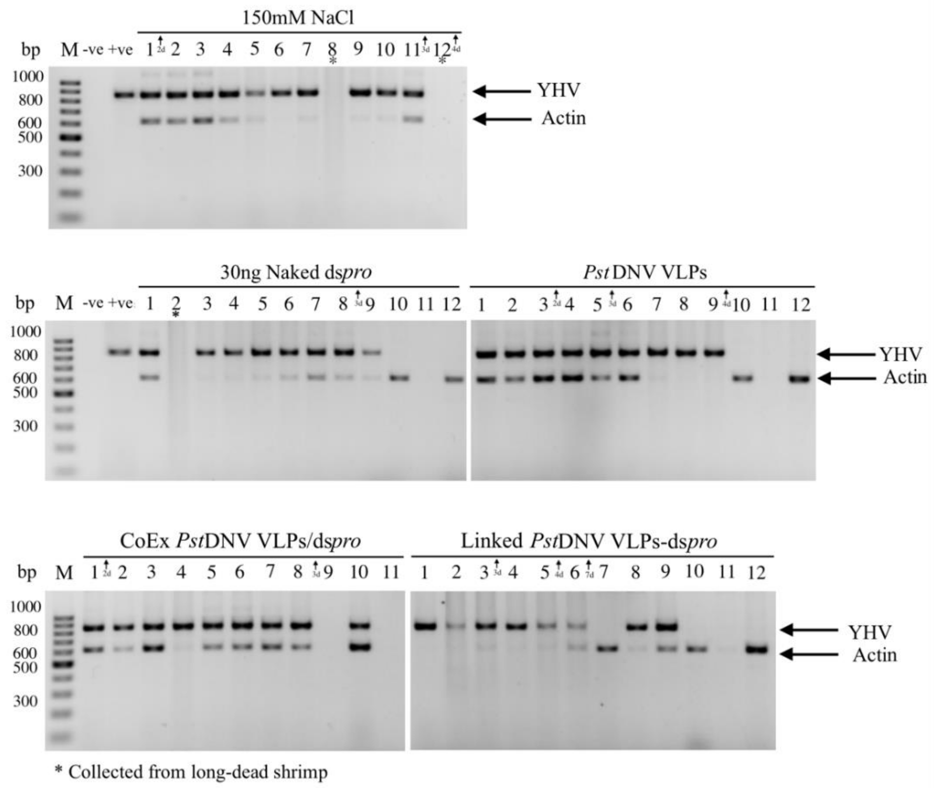

Figure S4. Confirmation of shrimp in all treatments infected with YHV**.** Agarose gel electrophoresis of RT-PCR products specific for YHV *helicase* from challenged shrimps. Host's *actin* gene was used as an internal control. Small arrows indicate the date on which each animal found dead following a virus challenge. Asterisk indicates sample collected from a long-dead shrimp. Lane M; GeneRuler 100 bp DNA Ladder (Thermo Fisher Scientific, Waltham, MA, USA), Lane +ve; positive control PCR, Lane -ve; negative control PCR.

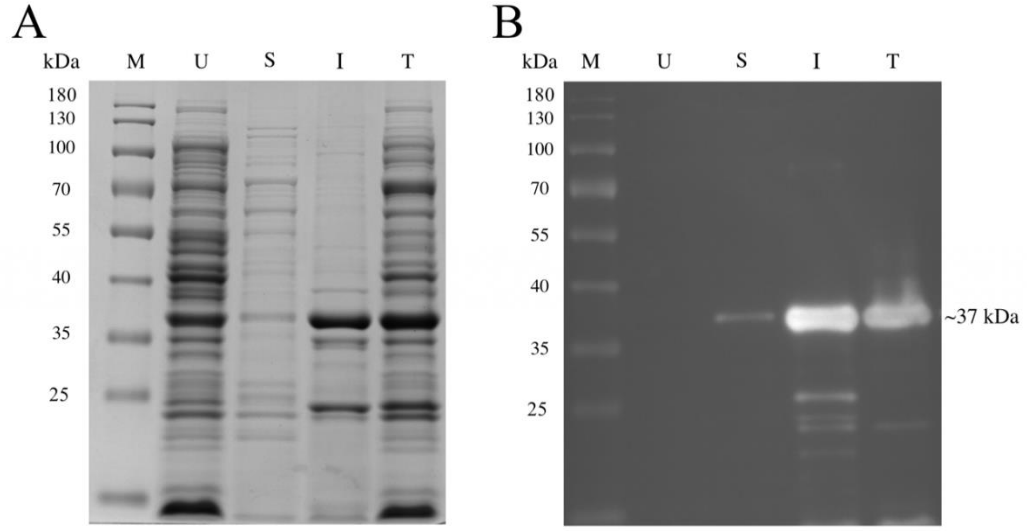

Figure S5. The solubility of recombinant Linked cp*Pst*DNV-ds*pro* in ENZhance lysis buffer. Induced bacteria (1 OD_{600nm}) were resuspended in ENZhance lysis buffer and incubated on ice for 30 min. After the bacterial suspension was centrifuged at 17,000 x*g* at 4 °C for 15 min, supernatant was taken (lane S) and cell debris was washed twice with sterile water (lane I). Both samples were then analyzed by SDS-PAGE (A) and western blotting (B). Lane M, PageRuler™ Prestained Protein Ladder (Thermo Fisher Scientific, Waltham, MA, USA), Lane U; total cell lysate of uninduced cell, Lane T, total cell lysate of induced cell.
